# Novel Alternative Splice Variants of Mouse *Cdk5rap2*

**DOI:** 10.1371/journal.pone.0136684

**Published:** 2015-08-31

**Authors:** Nadine Kraemer, Lina Issa-Jahns, Gerda Neubert, Ethiraj Ravindran, Shyamala Mani, Olaf Ninnemann, Angela M. Kaindl

**Affiliations:** 1 Institute of Cell Biology and Neurobiology, Charité –Universitätsmedizin Berlin, Berlin, Germany; 2 Department of Pediatric Neurology, Charité –Universitätsmedizin Berlin, Berlin, Germany; 3 Centre for Neuroscience, Indian Institute of Science, Bangalore, India; 4 Sozialpädiatrisches Zentrum (SPZ), Charité –Universitätsmedizin Berlin, Berlin, Germany; International Centre for Genetic Engineering and Biotechnology, ITALY

## Abstract

Autosomal recessive primary microcephaly (MCPH) is a rare neurodevelopmental disorder characterized by a pronounced reduction of brain volume and intellectual disability. A current model for the microcephaly phenotype invokes a stem cell proliferation and differentiation defect, which has moved the disease into the spotlight of stem cell biology and neurodevelopmental science. Homozygous mutations of the Cyclin-dependent kinase-5 regulatory subunit-associated protein 2 gene *CDK5RAP2* are one genetic cause of MCPH. To further characterize the pathomechanism underlying MCPH, we generated a conditional *Cdk5rap2 LoxP*/*hCMV Cre* mutant mouse. Further analysis, initiated on account of a lack of a microcephaly phenotype in these mutant mice, revealed the presence of previously unknown splice variants of the *Cdk5rap2* gene that are at least in part accountable for the lack of microcephaly in the mice.

## Introduction

Cyclin-dependent kinase-5 regulatory subunit-associated protein 2 (CDK5RAP2) has moved into the spotlight of neuroscience because of its central function in neural stem cell proliferation and thus brain development as well as its proposed role in mammalian brain evolution. Homozygous mutations in the *CDK5RAP2* gene cause autosomal recessive primary microcephaly type 3 (MCPH3) [1,2], a rare developmental disorder of the brain characterized by a pronounced reduction of brain volume, particularly of the neocortex, as well as intellectual disability (reviewed in [3–5]). One current model for the microcephaly phenotype of *CDK5RAP2* mutations invokes a premature shift from symmetric to asymmetric neural progenitor-cell divisions with a subsequent depletion of the progenitor pool and a reduction of the final number of neurons. In addition, we and others have proposed a reduction in cell survival [6,7].

Currently, no animal model exists that perfectly mimics MCPH. In contrast to the reported human phenotype, the *Hertwig’s anemia* mice (exon 4 inversion of the *Cdk5rap2* gene, deletion of a large part of the γ-tubulin ring complex (γTuRC) binding domain) not only have microcephaly, but also a hematopoietic phenotype (hypoproliferative anemia, leucopenia, predisposition to hematopoietic tumors) and defects in multiple organs [6]. Two further splice trap mutation mice (*Cdk5rap2* ^*RRF465*^, *Cdk5rap2* ^*RRU031*^) are not microcephalic [8]. A good animal model is crucial to linking cellular phenotypes of MCPH to physiological processes in the developing brain and understanding the mechanisms of neocortex development in this context.

In the present study, we report on novel splice variants of murine *Cdk5rap2*, which we identified in the course of the generation of a conditional mutant mouse (*Cdk5rap2 LoxP*/*hCMV Cre*). We were able to attribute the lack of an obvious microcephaly phenotype in these mice at least partially to previously unknown splice variants of the *Cdk5rap2* gene that are expressed in both mutant and wild-type mice.

## Material and Methods

### Construction of the *Cdk5rap2 LoxP* targeting vector

The conditional gene-targeting vector for the *Cdk5rap2 LoxP* ^*+/+*^ mice was produced from a mouse genomic library clone (*C57BL/6*). The targeting strategy was to conditionally delete exon 3 of *Cdk5rap2* and generate a subsequent stop codon at the beginning of exon 4 by using a Cre-LoxP strategy. The targeting vector was constructed by successive cloning of PCR products and contained 3.4 kb 5′ and 3.5 kb 3′ homology arms and a Neomycin selection cassette. Two LoxP sequences were introduced in similar orientation into intronic regions between exon 2 and 3 as well as between exon 3 and 4 to minimize disruption to the gene function, i.e., with positions about 320 bp before and about 2.2 kb at the end of exon 3. An FRT-Neo selection cassette with two flanking FRT sites for later removal by FLP recombinase [9] was inserted about 300 bp into intron 3 (first FRT site) and 16 bp (second FRT site) before the 3’ LoxP site (**S1 Fig**).

### Generation of *Cdk5rap2 LoxP+/- C57BL/6* embryonal stem cells

The linearized targeting construct was electroporated into *C57BL/6N* mouse embryonic stem cells (mESC) grown on feeders and selected using neomycin (6 positive in 372 screened clones). For the resultant clones, the correct insertion of the targeting construct into the genome was subsequently confirmed by PCR over the homologous recombination arm using external primers and further confirmed by Southern blot with Neo internal probe and with 5′ and 3′ external probes (**S1 Fig**, **S1–S3 Tables**).

### Generation of *Cdk5rap2 LoxP+/-* mice

The *Cdk5rap2 LoxP* ^*+/-*^ mouse line was established at the Institut Clinique de la Souris (ICS, llkirch, France) in accordance with the French law. One verified stem cell clone was selected for *C57BL/6* blastocysts injection, and mESC-derived chimeras [10] gave germline transmission. The resulting chimeric line was verified by PCR using external primers, further confirmed by Southern blot with Neo internal probe (**S1 Fig**) and crossed with a Flip *C57BL/6* deleter mouse to excise the FRT site-flanked Neo cassette on F1 progenies. The F1 animals were crossed with *C57BL/6* mice to generate F2 animals.

### Generation of conditional *Cdk5rap2 LoxP+/+ hCMV Cre+* mice

Conditional *Cdk5rap2 LoxP* ^*+/+*^ *hCMV Cre* ^*+*^ mice (cKO) were generated to obtain complete excision of the *Cdk5rap2* exon 3 and introduce a stop codon in exon 4. Breeding of *Cdk5rap2 LoxP* ^*+/+*^ mice with *hCMV Cre* ^*+*^ mice resulted in heterozygous *Cdk5rap2 LoxP* ^*+/-*^ *hCMV Cre* ^*+*^ mice which were then crossed with *Cdk5rap2 LoxP* ^*+/+*^ mice. The latter mice were further breed among each other. For experiments wildtype (WT), heterozygous knockout (het KO), and homozygous knockout (hom KO) mice were used. The corresponding genotypes are listed in **S4 Table**. Mice were kept and bred in an enriched environment in a SPF barrier at the animal facility FEM of the Charité - University Medicine Berlin, Germany and all experiments were carried out in accordance with the German ethic principles, approved by the State Office of Health and Social Affairs Berlin (Landesamt für Gesundheit und Soziales; LaGeSo; approval no. T0309/09 and G0113/08). The day of insemination was considered as embryonic day (E) 0 (E0), and the day of birth was designated as postnatal day (P) 0 (P0).

### Genotyping

Genomic DNA was isolated from tail sections by proteinase K digestion using standard methods, and genotyping was performed by PCR and subsequent agarose gel electrophoresis using four primer pairs (**S1 Fig, S5 and S6 Tables**).

### Mouse samples

Mice were sacrificed by decapitation (P0-P5) or cervical dislocation (adult). For conventional PCR, quantitative real-time PCR (qPCR) and Western blots, the cerebral cortex was quickly dissected from mice at P0 (n = 6 per group), snap-frozen and stored at -80°C for later RNA and protein extraction. For histological analysis, brains from mice at P0 and P5 (n = 6 per age) were dissected and immersed in 4% paraformaldehyde (PFA) in 0.12 M TPO4. Further brain fixation was performed in the same solution at 4°C for 1–2 h (embryonic brains) or overnight (postnatal brains). Brains were cryoprotected through overnight incubation in 10% sucrose 0.12 M TPO4 solution at 4°C followed by an overnight incubation in 20% sucrose 0.12 M TPO4. The brains were then immersed in a solution of 7.5% gelatin, 20% sucrose in 0.24 M TPO4 for 1 h at 37°C and subsequently embedded in a block with the same solution for 1 h at 4°C. The block was frozen in 2-metylbutan at -60°C and stored at -80°C. Coronal and sagittal sections of 10 μm thickness were cut on a cryostat and collected on Superfrost plus slides (R. Langenbrinck, Emmendingen, Germany).

### Human blood samples

mRNA derived from blood samples from controls were used in this study with approval from the local ethics committees of the Charité –University Medicine Berlin (approval no. EA1/212/08). An informed written consent was obtained from all control persons.

### Antibodies

We generated two peptide antibodies directed against mouse Cdk5rap2 in collaboration with Pineda antibody service (Berlin, Germany) as previously described [11]: (i) one antibody (N1) directed against a sequence of the N-terminal protein region NH2-DSGMEEEGALPGTLSGC-COOH; amino acids 2–18 of the Cdk5rap2 mouse sequence encoded by exon 1, accession no. NP_666102.2, and (ii) an antibody (A1) directed against a more centrally located protein region NH2-LKFEADVETPFQSDQHLEQSR-COOH; amino acids 705–725 of the full-length 1822 aa Cdk5rap2 mouse sequence encoded by exon 19, accession no. NP_666102.2. Both peptide sequences are unique for Cdk5rap2, and the specificity of the peptide antibody against Cdk5rap2 was previously tested by Western blot and on brain sections by peptide precompetition [11].

### RNA extraction and qPCR

Total RNA was extracted from tissue using TRI-Reagent (Sigma-Aldrich, Taufkirchen, Germany) according to the manufacturer’s recommendations. cDNA was prepared from 1 μg of RNA by reverse transcription using the ThermoScript RT-PCR System (Invitrogen, Karlsruhe, Germany) and a combination of oligo(dT)_20_ and random hexamer primers. For qPCR, 1 μl of 1:10 diluted cDNA was used as template. To specifically amplify and detect *Cdk5rap2* (WT allele, KO allele and novel spliced variant) and *Hprt* (Hypoxanthine-guanine phosphoribosyl-transferase, reference gene) cDNA, we designed the corresponding specific sets of sense and antisense primers and TaqMan probe using the GenScript real-time PCR (TaqMan) Primer Design online software (www.genscript.com) (**S7 Table**). Experiments (n = 6 per group, heterozygotes n = 5) were run in triplicate. PCR was performed in an Applied Biosystems 7500 Fast Real-time PCR System (Applied Biosystems Inc., Norwalk, CT, USA) in 96-well microtiter plates using a final volume of 13 μl. The reaction mixture consisted of 1x TaqMan Universal PCR Master Mix, No AmpErase UNG (Roche, Branchburg, NJ, USA), 385 nM primer F, 385 nM primer R, 230 nM probe, and 1 μl template cDNA. Amplification was performed with the thermal profile of 50°C for 2 min, initial denaturation at 95°C for 10 min, followed by 40 cycles of denaturation at 95°C for 15 sec and a combined primer annealing/extension step at 60°C for 1 min, during which the fluorescence signal was acquired. Ct values were calculated using the 7500 Fast System SDS Software (Applied Biosystems Inc.), and further statistical calculations were performed on Microsoft Excel (Microsoft Corporation, Bellevue, WA, USA) and GraphPad Prism 5 Software (GraphPad Software Inc., La Jolla, CA, USA). The 2^-ΔΔCt^ method was applied for the quantification of the relative expression of the *Cdk5rap2* mRNA using the reference gene *Hprt* as the endogenous control for normalization.

### Sequencing of murine and human *CDK5RAP2* cDNA

cDNA samples were prepared from cortex of P0 mice and from human fibroblasts as described above. Primers used for PCR are given in **S8 Table.** The PCR products were separated on a 1.5% agarose gel, and bands were excised, purified, and cloned into a pCR2.1-TOPO TA vector using the TOPO TA cloning kit (Invitrogen) according to the protocol provided by the manufacturer. Samples were sequenced using M13 forward and reverse primers. The full length *Cdk5rap2* mRNA sequence, accession no. NM_145990.3, was used as reference sequence.

### Rapid amplification of cDNA ends (RACE)

To identify putative N-terminally shorter *Cdk5rap2* variants, we applied the 5’ RACE-PCR technique (Roche) according to the protocol provided by the manufacturer. Specific primers used for nested PCR are given in **S9 Table**. The PCR products were separated on a 1.5% agarose gel and further processed for sequencing as described above.

### Protein extraction procedure and Western blot

Protein extracts for Western blots were isolated from tissues by homogenization in RIPA buffer containing 1 mM PMSF (Sigma-Aldrich) and 1 protease inhibitor cocktail tablet (Complete Mini; Roche Diagnostics, Mannheim, Germany), 20 min incubation on ice and centrifugation at 4°C for 10 min at 3,000 g and for 20 min at 16,000 g. Protein concentrations were determined using a bicinchoninic acid (BCA) based assay, according to the instructions of the manufacturer (BCA Protein Assay Kit; Pierce Biotechnology, Rockford, IL, USA). Protein extracts (30 μg per sample) were denaturated in Laemmli sample loading buffer at 95°C for 5 min, separated by sodium dodecyl sulphate polyacrylamide gel electrophoresis (SDS-PAGE) and electrophoretically transferred in transfer buffer in a semi-dry fashion using Trans-Blot SD Semi-Dry transfer cell (Bio-Rad, Munich, Germany) onto nitrocellulose membrane (Bio-Rad). The membranes were incubated for 1 h at room temperature (RT) in blocking buffer (TBS-T 1x with 5% bovine serum albumin (BSA)), rinsed three times with TBS-T 1x for 8 min each at RT on a shaker and then incubated overnight at 4°C with rabbit anti-Cdk5rap2 (N1 or A1) (1:500) and mouse anti-beta actin (1:10,000; Sigma-Aldrich) antibodies. After incubation with the corresponding secondary antibodies donkey anti-rabbit (1:2000; Amersham Biosciences, Freiburg, Germany) and goat anti-mouse (1:10,000; Dako, Hamburg, Germany), the immunoreactive proteins were visualized using a technique based on a chemiluminescent reaction. The gel pictures were obtained on photographic films (Amersham Hyperfilm enhanced chemiluminescence (ECL); GE Healthcare, Freiburg, Germany). Western blot experiments were run in triplicate.

### Immunohistology and imaging

Cryostat sections were air-dried briefly prior to rinsing in phosphate buffered saline (PBS 1x) for 10 min and in staining buffer (0.2% gelatin, 0.25% Triton X-100 in PBS 1x) for 20 min. In a 30 min blocking step, sections were incubated in 10% donkey normal serum (DNS) in staining buffer at RT. Sections were incubated overnight at RT with primary antibodies in the staining buffer containing 10% DNS followed by an incubation with the corresponding secondary antibodies for 2 h at RT. Nuclei were labeled with 4’,6-diamidino-2-phenylindole (DAPI; 1:1000; Sigma-Aldrich). Fluorescently labeled sections were analyzed and imaged by a fluorescent Olympus BX51 microscope with the software Magnafire 2.1B (2001) (Olympus, Hamburg, Germany), and confocal microscopy images were taken by an lsm5exciter Zeiss confocal microscope with the software Zen (version 2009, Zeiss, Jena, Germany). All images were processed using Adobe Photoshop (Adobe Systems, San José, CA, USA).

### Blood counts

Blood samples were obtained from mice following cervical dislocation through intracardial puncture and slow aspiration. Blood slides were prepared and blood cells were counted manually.

### Cranial MRI analysis

Cranial magnetic resonance imaging (cMRI) was performed using a 7 Tesla rodent scanner (Pharmascan 70 ⁄ 16, Bruker BioSpin, Ettlingen, Germany). For imaging, a ^1^H-RF quadrature-volume resonator with an inner diameter of 20 mm was used. Data acquisition and image processing were carried out with the Bruker software Paravision 4.0. During the examinations mice were placed on a heated circulating water blanket to ensure constant body temperature of 37°C. Anaesthesia was induced with 3% and maintained with 1.5–2.0% isoflurane (Forene, Abbot, Wiesbaden, Germany) delivered in a O_2_ / N_2_O mixture (0.3 / 0.7 l/min) via a facemask under constant ventilation monitoring (Small Animal Monitoring & Gating System, SA Instruments, Stony Brook, New York, USA). The animals were placed on a custom-built animal holder. For imaging the mouse brain in axial and sagittal orientation, a T2-weighted 2D turbo spin-echo sequence with a RARE factor of 8 and 4 averages was used. Imaging parameters: (i) axial TR / TE = 4059 / 36 ms, 35 axial slices with slice thickness of 0.5 mm, field of view of (FOV) 2.60 x 2.60 cm, matrix size 256 x 256, (ii) sagittal TR / TE = 4200 / 36 ms, 20 sagittal slices with slice thickness of 0.5 mm, FOV 2.60 x 2.60 cm, matrix size 256 x 256. cMRI data processing was performed with Image J software. Section areas and brain volumes were calculated (n = 4–6 per group).

## Results

### Generation and verification of conditional *Cdk5rap2 LoxP hCMV* Cre mutant mouse

**Fig 1:**
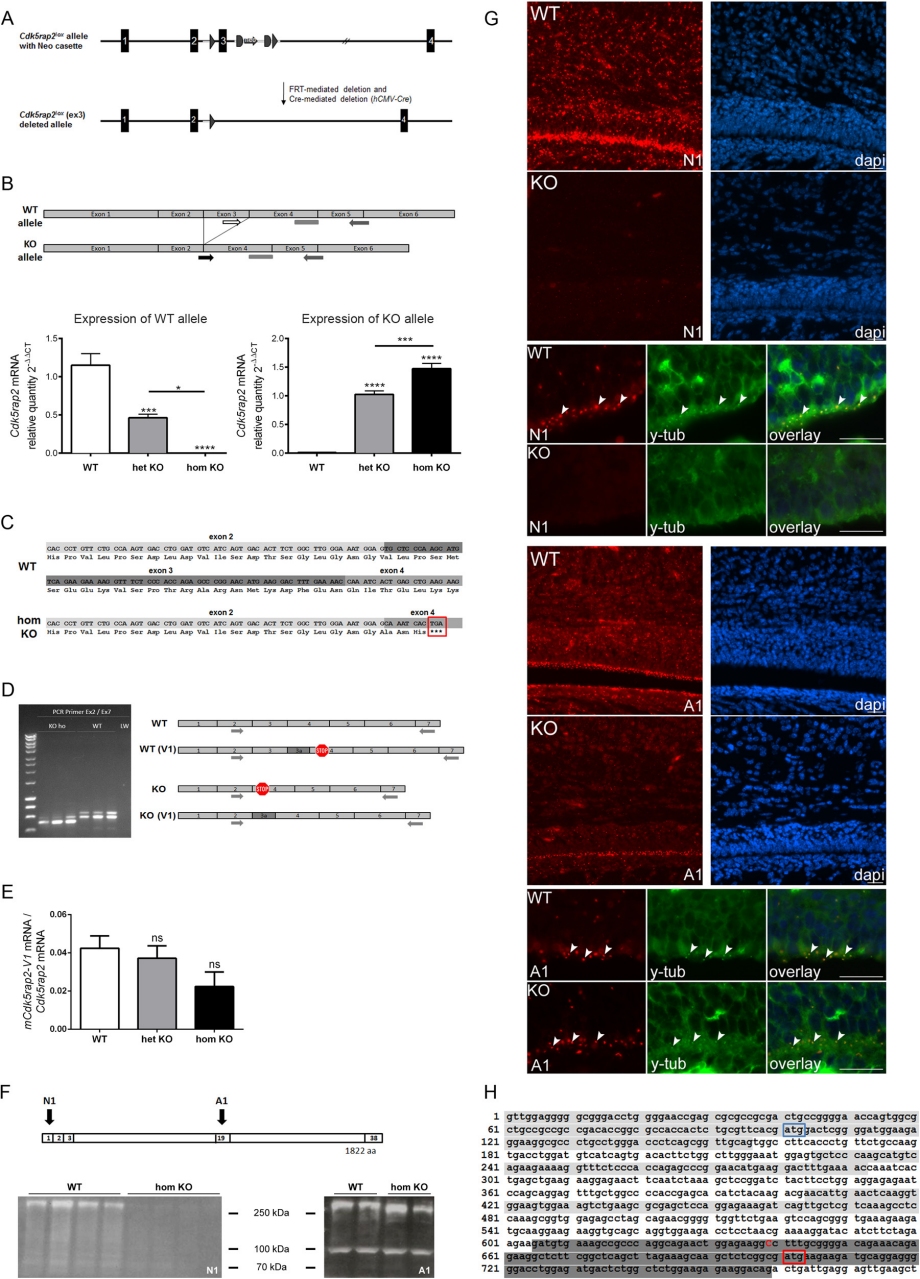
Conditional *Cdk5rap2* KO mouse and novel *mCdk5rap2* splice variants. (A) *Cdk5rap2* ^*lox*^ construct before and after Cre-mediated deletion of exon 3. (B) *Cdk5rap2* WT and KO allele expression in the neocortex of WT, het KO, and hom KO mice at P0. The WT allele is not expressed in hom KO mice; the KO allele is not expressed in WT mice (qPCR, n = 6 per group, one-way ANOVA, p<0.0001; Bonferroni’s Multiple Comparison Test). Position of forward primers specific for the WT and the KO allele, respectively, and the common reverse primer and probe are depicted. (C) Sanger sequencing results of PCR products from exon 2 to exon 7 of WT and hom KO cDNA confirmed the correct excision of exon 3 in hom KO mice, resulting in a frame shift and a premature stop codon (red box). (D) Identification of a novel *mCdk5rap2* splice variant (*mCdk5rap2-V1*) through gel electrophoresis and sequencing of PCR products from exon 2 to exon 7 of WT and hom KO cDNA. In hom KO mice, the additional 71 nucleotides will abolish the frameshift and stop codon introduced through excision of exon 3. In WT mice the additional 71 nucleotides will lead to a frameshift and a premature stop codon resulting in a truncated 85 aa protein. (E) *mCdk5rap2-V1* mRNA expression in the neocortex of WT, het KO, and hom KO mice at P0 (qPCR, n = 6 per group, one-way ANOVA, p = 0.1314; Bonferroni’s Multiple Comparison Test). (F) Western blot analysis using the N1 antibody revealed a strong reduction of protein levels below detection levels in hom KO in comparison to WT neocortex at P0, while multiple bands were identified with a reduction in the size of the largest (about 250 kDa) band by about 10 kDa when using the A1 antibody. Binding sites of anti-Cdk5rap2 antibodies N1 and A1 are depicted, numbers refer to exons encoding the corresponding protein regions. (G) Immunostaining using antibodies directed against Cdk5rap2 (red) and the centrosome marker y-tubulin (green) of coronal murine hom KO and WT brain sections at P0; nuclei are stained with DAPI (blue). Overview immunofluorescence pictures and higher magnification images of the subventricular (SVZ) and the ventricular (VZ) zone; arrowheads indicate examples for centrosomes which are co-stained with Cdk5rap2 and y-tubulin; scale bars 20 μm. When applying the N1 antibody, high Cdk5rap2 signal density is present within the neocortex and SVZ/VZ in WT mice, while this is lacking in hom KO mouse brains. The A1 antibody, however, produces Cdk5rap2 immunopositivity in both the hom KO and WT neocortex and SVZ/VZ with a high signal density in the in the WT mice and a similar pattern with only a slight decrease of staining density and intensity in hom KO mice. (H) Excerpt of *Cdk5rap2* mRNA sequence (NM_145990.3, exons 1, 3, and 5 are highlighted in light grey, exon 7 in dark grey) with known start codon in exon 1 (blue box) and alternative start codon in exon 7 (red box) as well as putative sequence start of shorter variant *Cdk5rap2-V2* (red upper case C). ns, not significant, *p<0.05, **p<0.01, ***p<0.001, ****p<0.0001.

We generated mutant *Cdk5rap2* mice on a *C57BL/6N* background by introducing a LoxP site flanking exon 3 of the *Cdk5rap2* gene and, following verification steps, breeding the resulting *Cdk5rap2 LoxP* ^*+/-*^ mice with *hCMV* Cre mice (**Fig 1A**, see Material and Methods and **S1 Fig** for details). The conditional *Cdk5rap2 LoxP* ^*+/+*^ *hCMV Cre* ^*+*^ mice (cKO) were generated to obtain complete excision of the Cdk5rap2 exon 3 and thereby introduce a frameshift and a subsequent stop codon in exon 4. Heterozygous *Cdk5rap2 LoxP* ^*+/-*^ *hCMV Cre* ^*+*^ mice were crossed with *Cdk5rap2 LoxP* ^*+/+*^ mice, followed by an inbreeding of the resulting mice. For experiments wildtype (WT), heterozygous knockout (het KO), and homozygous knockout (hom KO) mice were used (for corresponding genotypes, see **S4 Table**).

### Lack of phenotype of *Cdk5rap2* mutant mouse

We did not detect a microcephaly phenotype when analyzing the animals through clinical examination, body and brain weight measurements, cMRI analysis, and histological assessment of their brains (**S2 Fig**). cMRI analysis of brains of hom KO and WT mice at P56 did not reveal a significant reduction of brain or neocortex volume.

### Identification of a novel murine *Cdk5rap2* splice variant (*mCdk5rap2-V1*)

To further address this point despite the known background dependency of *Cdk5rap2* mutant mice [6] and thus to verify the correct generation of the planned mutant construct, we performed quantitative real-time PCR (qPCR) analysis using forward primers specific for the WT and the KO allele, respectively, in combination with a common reverse primer and probe. The WT specific primer binds to a sequence in exon 3, while the KO specific primer binds only when exon 3 is deleted completely as it recognizes a sequence composed of the 3’-end of exon 2 and the 5’-end of exon 4 (**Fig 1B**, **S7 Table**). The qPCR confirmed the correct excision of exon 3 in the hom KO mice as *Cdk5rap2* mRNA could only be detected with the KO, but not with the WT specific primer pairs. In line with this, *Cdk5rap2* mRNA could only be detected with the WT but not the KO specific primer pair in the WT, and with both primer pairs in the het KO samples. *Cdk5rap2* mRNA levels were increased in het and hom KO compared to WT (**Fig 1B**). In addition, we Sanger sequenced cDNA from WT and hom KO mice. The sequencing results also confirmed the correct excision of exon 3 in the hom KO, leading to a frameshift and a premature stop codon in exon 4 (**Fig 1C**). In the course of the sequencing procedure we performed PCRs to enrich the sequence fragment of interest using forward and reverse primers that bind to exon 2 and 7, respectively. Separation of the respective PCR products revealed an additional band in hom KO and WT which was ~70 bp longer than the expected 586 bp for WT and 518 bp for hom KO, respectively (**Fig 1D**). This additional band was also detected using primers binding in exon 1 (F) and exon 11 (R) (data not shown). We therefore Sanger sequenced the additional PCR product bands following cloning into TOPO-TA plasmids. We identified a novel murine *Cdk5rap2* splice variant (*mCdk5rap2-V1*) which contains an additional exon (exon 3a) of 71 nucleotides. Exon 3a lies between exon 3 and 4, and its expression in the cKO mouse will abolish the frameshift and stop codon introduced through excision of exon 3 (**Fig 1D**). In WT mice, translation of *mCdk5rap2-V1* results in a truncated 85 aa protein. This additional exon 3a is not present in human *CDK5RAP2* mRNA (data not shown).

### Quantification of *mCdk5rap2*-*V1* mRNA

To quantify the relative mRNA amount of the novel splice variant *mCdk5rap2-V1* in WT, het KO, and hom KO mice, we performed qPCR using the primer and probe set of WT and KO allele specific and common primers as described above. In addition, we applied a *mCdk5rap2*-*V1* specific forward primer which binds to a sequence in exon 3a. The fraction of *mCdk5rap2*-*V1* mRNA relative to the *Cdk5rap2* mRNA was very low (4.2% in WT, 3.7% in het KO, 2.2% in hom KO) and was not increased in the het or hom KO mice (**Fig 1E**).

### Second novel murine *Cdk5rap2* splice variant (*mCdk5rap2-V2*)

Cdk5rap2 protein levels were below the detection level when assessed through immunohistological and Western blot analysis of hom KO mouse brains using the N-terminal antibody (N1) binding to amino acids 2–18 of the Cdk5rap2 mouse protein sequence (**Fig 1F and 1G**). While this is in line with a nonsense-mediated mRNA decay or rapid degradation of the putative truncated Cdk5rap2 protein, we did detect Cdk5rap2 with an antibody (A1) directed towards a more centrally located Cdk5rap2 site, both in immunohistological analysis and in Western blots (**Fig 1F and 1G**). Sequence analysis of the *Cdk5rap2* gene indicated an ATG site flanked by a Kozak consensus sequence in exon 7 of the *Cdk5rap2* gene to possibly serve as an additional start codon. The existence of a shorter *Cdk5rap2* variant with the translation starting in exon 7 would result in a 1622 aa Cdk5rap2 variant and might explain why low level of or no Cdk5rap2 protein is detected in the hom KO when using an antibody against the N-terminus, while a product can still be identified through a second antibody directed towards amino acids 705–725 of the full-length 1822 aa Cdk5rap2 mouse sequence. To further address this hypothesis of a shorter Cdk5rap2 variant lacking the N-terminus, we performed 5’ RACE-PCR analysis using specific primers for the nested PCR directed subsequently against exons 14, 12, and 9. The PCR products yielded three dominant bands which were sequenced following cloning into a TOPO-TA plasmid. Sanger sequencing identified several of the clones starting at base pair position 639 of the *Cdk5rap2* mRNA reference sequence, which is close to the presumptive alternative start codon in exon 7 at position 701 (**Fig 1H**). The latter results thus indicate the existence of a second, shorter Cdk5rap2 variant lacking the N-terminus.

## Discussion

**Fig 2:**
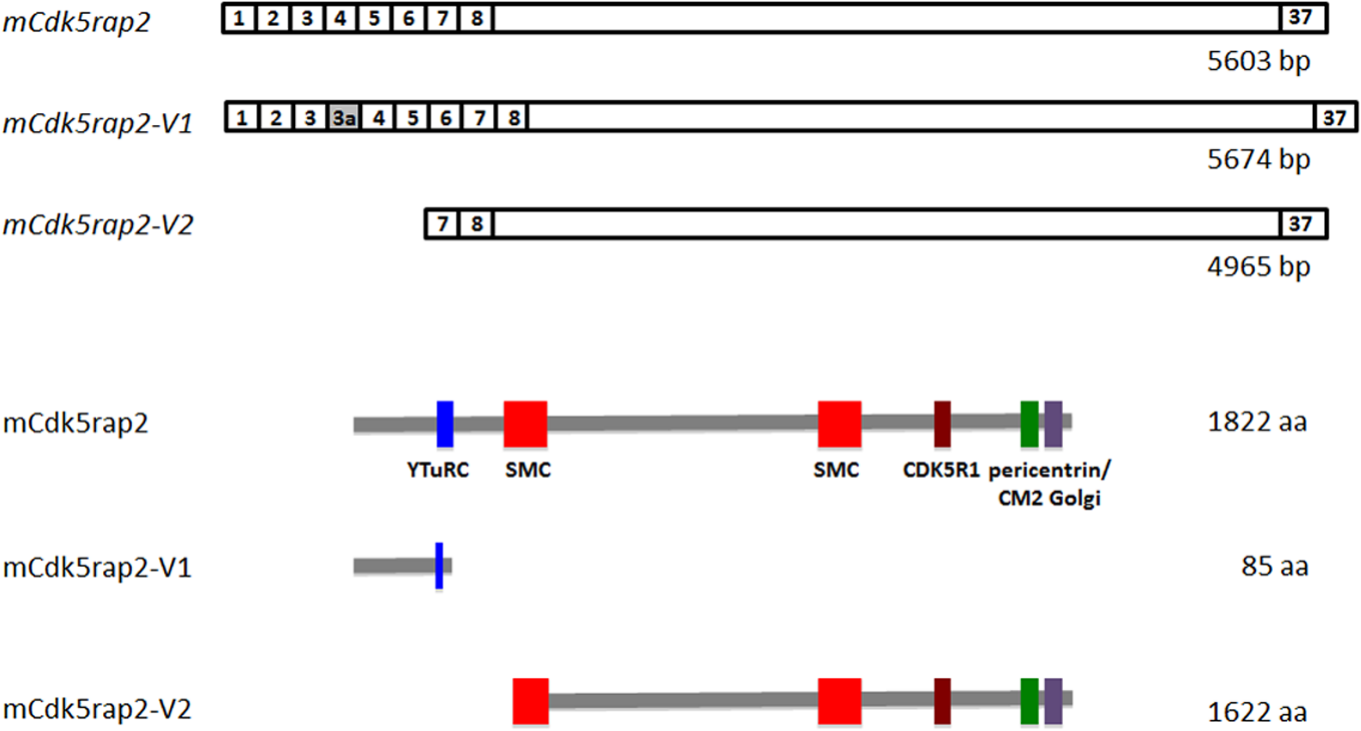
*mCdk5rap2* splice variants. Schematic representation of mCdk5rap2, mCdk5rap2-V1, and mCdk5rap2-V2 mRNA (top) and protein (bottom).

The lack of a phenotype of conditional *Cdk5rap2* knockout mice lead us to analyze *Cdk5rap2* mRNA in mutant (hom KO) and wild-type (WT) mice in detail. We thereby identified one mRNA variant (*mCdk5rap2*-*V1*) with an additional exon 3a, which is expressed both in the WT as well as in the hom KO mice. Given that *mCdk5rap2-V1* is expressed only at very low levels and is not upregulated in the mutant mice, it is unlikely that this variant is solely responsible for the rescue of the phenotype in the *Cdk5rap2* cKO mice. Moreover, we did not detect immunopositivity in hom KO cortical sections when applying the N-terminal antibody N1, which should recognize *mCdk5rap2*-*V1*. In addition to this, the detection of Cdk5rap2 in hom KO mice cortex with the more C-terminal antibody A1 indicates the existence of an additional variant. In this regard, our data further suggest a second *Cdk5rap2* mRNA variant (*mCdk5rap2*-*V2*) which lacks the N-terminal part of the full length *Cdk5rap2* mRNA sequence from bp 1 to 638 (exon 1 to 6) and uses an alternative start codon in exon 7. Since in WT mice translation of *mCdk5rap2-V1* results in a truncated 85 aa protein it is most likely that this variant is physiologically redundant (**Fig 2**). The suggested variant *mCdk5rap2-V2* lacks the γTuRC binding domain, which has been reported to be important for the γTuRC attachment to the centrosome and therefore for the microtubule organizing function of the centrosome [12]. However, *mCdk5rap2-V2* localizes to the centrosome in mouse neocortex, as shown in immunohistological stainings. This allows speculation that potentially targeting Cdk5rap2 to the centrosome is not exclusively dependent of the yTuRC site but rather on interaction with other proteins such as pericentrin. The functional significance of these variants as well as those predicted in genome datasets (**S3 Fig**) and the human variants (**S4 Fig**) will need to be addressed in further functional studies.

## Supporting Information

S1 Fig Generation and verification of *Cdk5rap2 LoxP* ^*+/-*^ allele. (A) Schematic representation of the targeting vector. Homologous recombination into the *Cdk5rap2* wildtype allele of mouse embryonic stem cells resulted into the displayed genotype. A correctly targeted ESC clone was injected into blastocyst stage embryos to generate chimeric mice. Chimeras were bred with FLIP (FLP) transgenic mice to generate *Cdk5rap2* LoxP mice lacking the Neo cassette. Recombinase recognition sites: FRT—Flp recombinase; loxP—Cre recombinase. The correct insertion of the targeting construct into the genome was confirmed by (B) PCR screening of clones, (C) Neo Southern, and (D) external Southern. (E) Genotyping of mice (see **S1 Table** for primer sequences).
(JPG)

S2 Fig Characterization of conditional *Cdk5rap2* knockout mouse. Conditional *Cdk5rap2* knockout (cKO) mice were generated by breeding *Cdk5rap2 LoxP* ^*+/+*^ mice with *hCMV Cre* ^*+/-*^ mice. P0 and adult (P56) hom KO mice had normal (A) body weight and (B) brain weight when compared to WT controls. (C) Magnetic resonance imaging (MRI) analysis of brains of hom KO and WT mice at P56 revealed no significant difference in brain volume (n = 3–7 per group). There was a slight reduction of neocortex volume (n = 3–4 per group). (D) Hom KO mice had normal blood counts at P56 when compared to WT control mice. Abbreviations: WBC, white blood count; RBC, red blood count; HGB, hemoglobin; HCT, hematocrit; MCV, mean corpuscular volume of erythrocytes; MCH, mean corpuscular hemoglobin of erythrocytes; MCHC, mean corpuscular hemoglobin concentration; PLT, platelet counts. Students t-test; values represent mean ± S.E.M.; *p<0.05, **p<0.01, ***p<0.001.
(JPG)

S3 Fig Known and predicted *mCdk5rap2* transcript variants in genome datasets. In addition to the *mCdk5rap2* RefSeq NM_145990.3 (Ensembl transcript ID: ENSMUST00000144099) the available genome databases (NCBI, Ensembl, MGI) list several *mCdk5rap2* variants, which have not been confirmed so far. The NCBI dataset comprises 10 predicted transcript variants (X1 –X10), annotated using the gene prediction method Gnomon and thus supported by mRNA and EST evidence. In all cases, the support level by ESTs is very low as only one or maximal two ESTs are available for altered regions. Ensemble lists 5 additional transcript variants: two without an open reading frame, hence not protein-encoding, one predicted to undergo nonsense mediated decay, and two which are predicted to be protein coding. All of these variants have a low transcript support level according to the Ensembl definition. None of these predicted variants is similar to *mCdk5rap2-V1* or *mCdk5rap2-V2*. Given the large size of the *mCdk5rap2* gene, it is most likely that more transcript variants exist as already confirmed for the human *CDK5RAP2* (**S4 Fig**). Further investigation will be needed to compile the existing variants which might be helpful to understand the diverse physiological functions of Cdk5rap2 in different tissues.Schematic diagram of *mCdk5rap2* transcript variants. Exon numbering is according to the *mCdk5rap2* RefSeq NM_145990.3; schematic exons do not reflect the actual exon size. Changes in predicted variants compared to the RefSeq NM_145990.3 are marked with red for additional exons, blue for exons containing additional base pairs, and green for shortened exons missing some base pairs.
(JPG)

S4 Fig Human *CDK5RAP2* transcript variants in genome datasets. Overview about all human *CDK5RAP2* transcript variants listed in genome databases (NCBI, Ensembl, MGI) so far. Schematic diagram of *CDK5RAP2* transcript variants. Exon numbering is according to the *CDK5RAP2*, transcript variant 1 RefSeq NM_018249.5; schematic exons do not reflect the actual exon size. Changes in variants compared to the RefSeq NM_018249.5 are marked with red for additional exons, blue for exons containing additional base pairs, green for shortened exons missing some base pairs, and with ‘in’ for retained introns.
(JPG)

S1 Table Primer sequences used for PCR validation of positive ESC clones. (DOCX)

S2 Table Neo Southern: Digestions used to validate the 5’ and 3’ insertion. (DOCX)

S3 Table External Probe Southern: Digestions used to validate with 5’ and 3’ probes. (DOCX)

S4 Table Cdk5rap2 mutant mice used in experiments. (DOCX)

S5 Table Primer sequences for genotyping. (DOCX)

S6 Table PCR fragments expected size (bp). (DOCX)

S7 Table Primer and probe sequences for qPCR. (DOCX)

S8 Table Primer sequences for sequencing of human *CDK5RAP2*. (DOCX)

S9 Table Primer sequences for 5’-RACE. (DOCX)
