# Supplementary figures and images for: Novel Alternative Splice Variants of Mouse Cdk5rap2
Source: PLoS One. 2015 Aug 31;10(8):e0136684. doi: 10.1371/journal.pone.0136684 (PMC4556188; doi:10.1371/journal.pone.0136684)

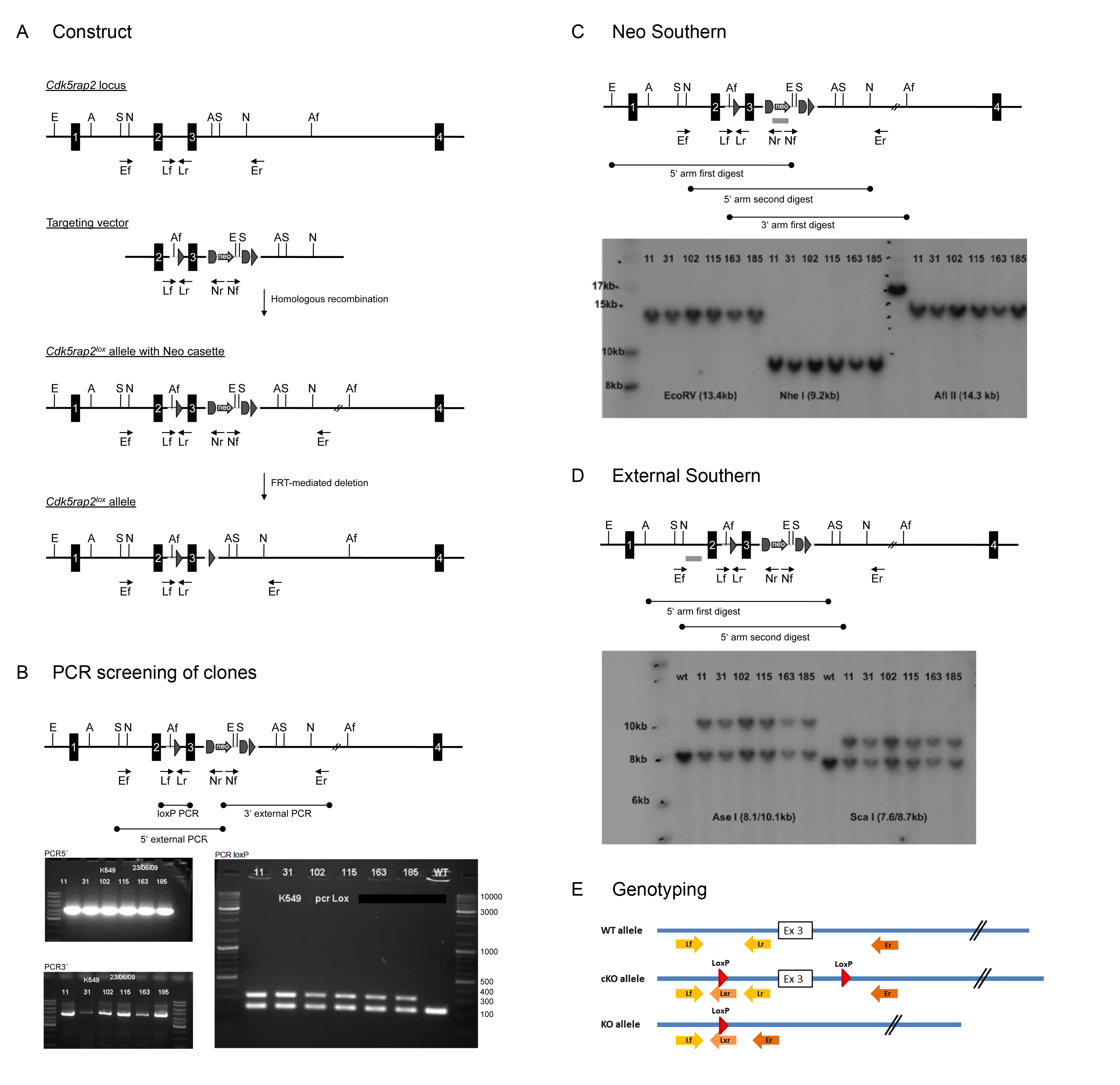

Supplement: S1 Fig — (A) Schematic representation of the targeting vector. Homologous recombination into the Cdk5rap2 wildtype allele of mouse embryonic stem cells resulted into the displayed genotype. A correctly targeted ESC clone was injected into blastocyst stage embryos to generate chimeric mice. Chimeras were bred with FLIP (FLP) transgenic mice to generate Cdk5rap2 LoxP mice lacking the Neo cassette. Recombinase recognition sites: FRT—Flp recombinase; loxP—Cre recombinase. The correct insertion of the targeting construct into the genome was confirmed by (B) PCR screening of clones, (C) Neo Southern, and (D) external Southern. (E) Genotyping of mice (see S1 Table for primer sequences). (JPG) [file pone.0136684.s001.jpg]

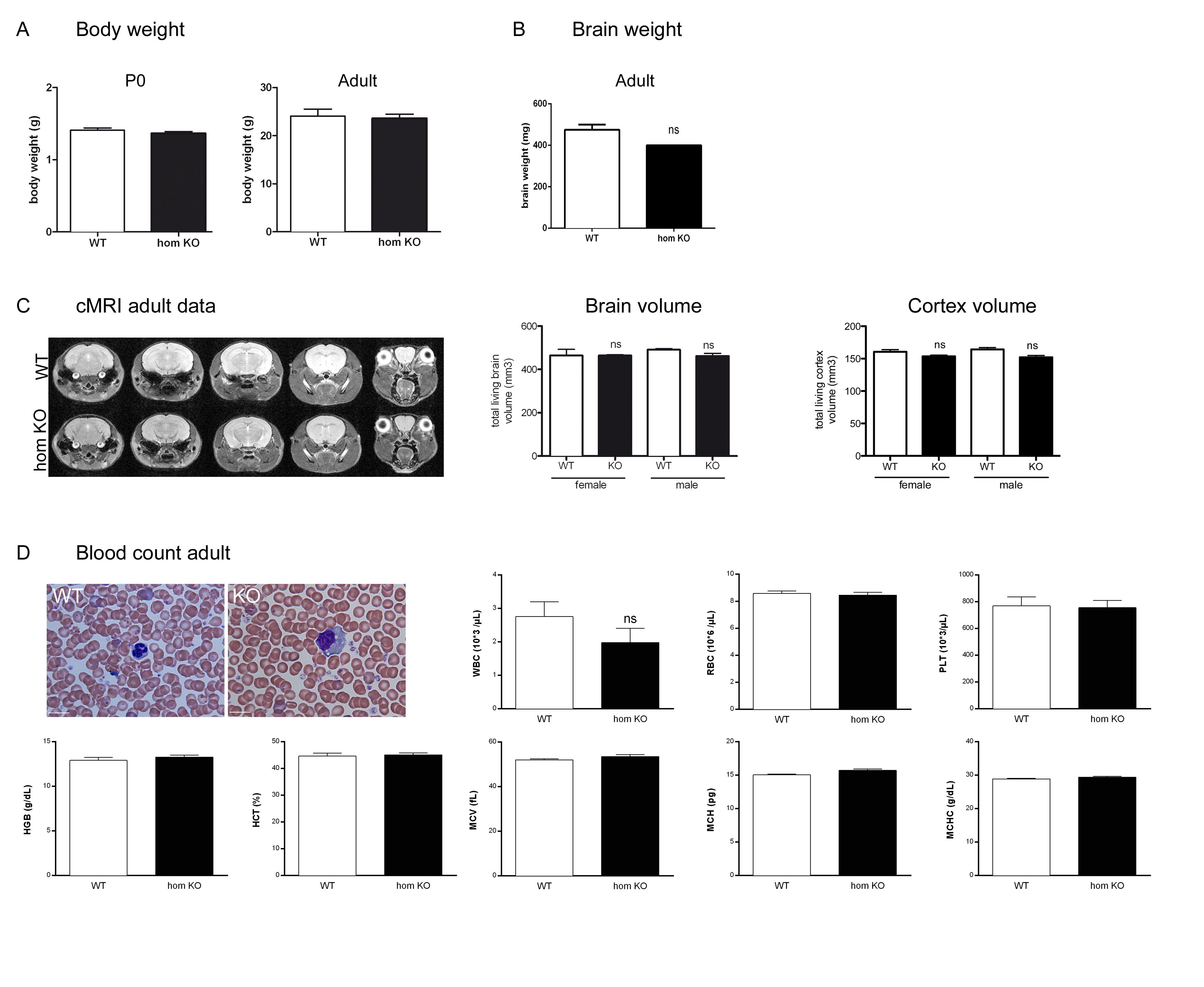

Supplement: S2 Fig — Conditional Cdk5rap2 knockout (cKO) mice were generated by breeding Cdk5rap2 LoxP +/+ mice with hCMV Cre +/- mice. P0 and adult (P56) hom KO mice had normal (A) body weight and (B) brain weight when compared to WT controls. (C) Magnetic resonance imaging (MRI) analysis of brains of hom KO and WT mice at P56 revealed no significant difference in brain volume (n = 3–7 per group). There was a slight reduction of neocortex volume (n = 3–4 per group). (D) Hom KO mice had normal blood counts at P56 when compared to WT control mice. Abbreviations: WBC, white blood count; RBC, red blood count; HGB, hemoglobin; HCT, hematocrit; MCV, mean corpuscular volume of erythrocytes; MCH, mean corpuscular hemoglobin of erythrocytes; MCHC, mean corpuscular hemoglobin concentration; PLT, platelet counts. Students t-test; values represent mean ± S.E.M.; *p<0.05, **p<0.01, ***p<0.001. (JPG) [file pone.0136684.s002.jpg]

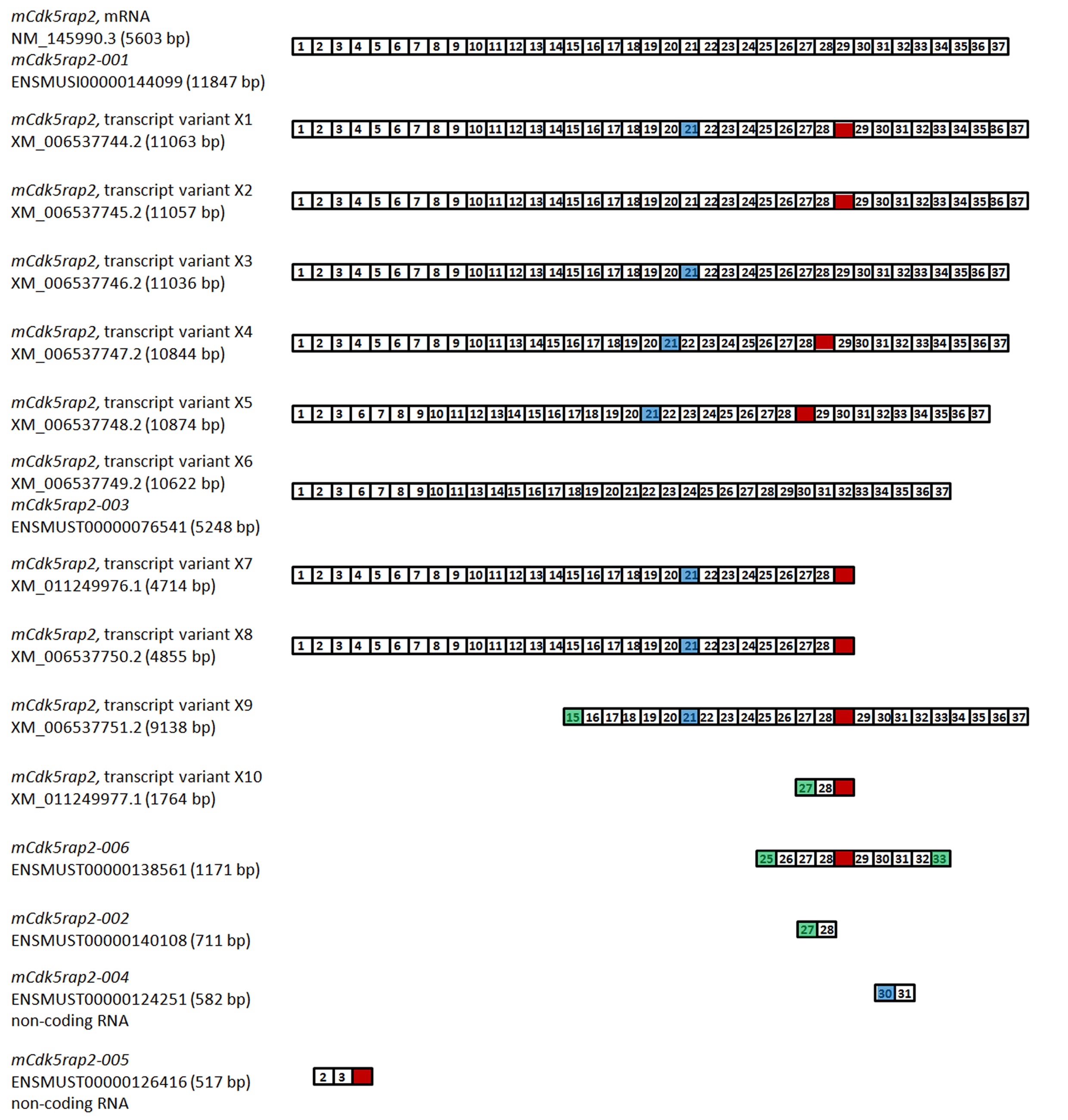

Supplement: S3 Fig — In addition to the mCdk5rap2 RefSeq NM_145990.3 (Ensembl transcript ID: ENSMUST00000144099) the available genome databases (NCBI, Ensembl, MGI) list several mCdk5rap2 variants, which have not been confirmed so far. The NCBI dataset comprises 10 predicted transcript variants (X1 –X10), annotated using the gene prediction method Gnomon and thus supported by mRNA and EST evidence. In all cases, the support level by ESTs is very low as only one or maximal two ESTs are available for altered regions. Ensemble lists 5 additional transcript variants: two without an open reading frame, hence not protein-encoding, one predicted to undergo nonsense mediated decay, and two which are predicted to be protein coding. All of these variants have a low transcript support level according to the Ensembl definition. None of these predicted variants is similar to mCdk5rap2-V1 or mCdk5rap2-V2. Given the large size of the mCdk5rap2 gene, it is most likely that more transcript variants exist as already confirmed for the human CDK5RAP2 (S4 Fig). Further investigation will be needed to compile the existing variants which might be helpful to understand the diverse physiological functions of Cdk5rap2 in different tissues.Schematic diagram of mCdk5rap2 transcript variants. Exon numbering is according to the mCdk5rap2 RefSeq NM_145990.3; schematic exons do not reflect the actual exon size. Changes in predicted variants compared to the RefSeq NM_145990.3 are marked with red for additional exons, blue for exons containing additional base pairs, and green for shortened exons missing some base pairs. (JPG) [file pone.0136684.s003.jpg]

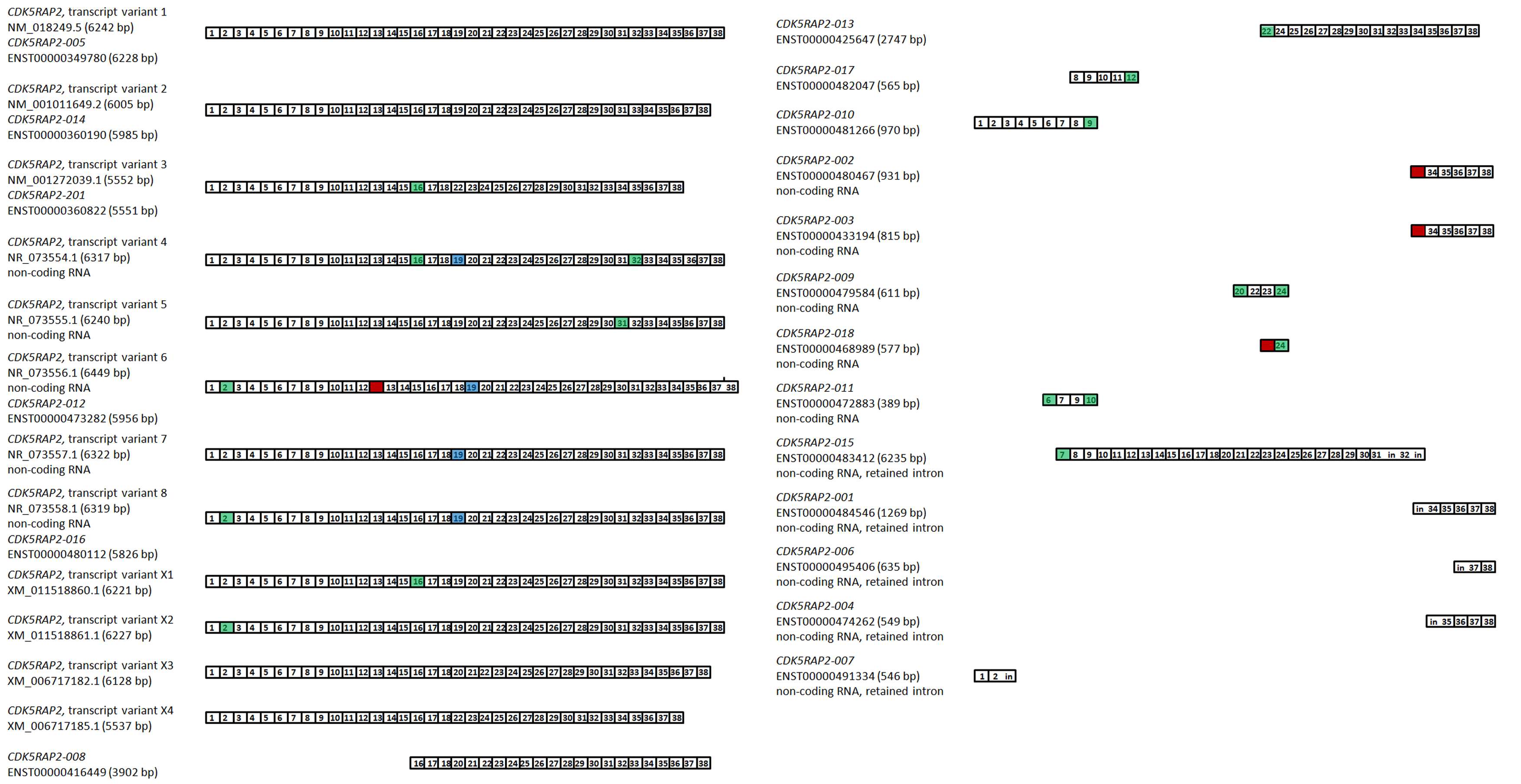

Supplement: S4 Fig — Overview about all human CDK5RAP2 transcript variants listed in genome databases (NCBI, Ensembl, MGI) so far. Schematic diagram of CDK5RAP2 transcript variants. Exon numbering is according to the CDK5RAP2, transcript variant 1 RefSeq NM_018249.5; schematic exons do not reflect the actual exon size. Changes in variants compared to the RefSeq NM_018249.5 are marked with red for additional exons, blue for exons containing additional base pairs, green for shortened exons missing some base pairs, and with ‘in’ for retained introns. (JPG) [file pone.0136684.s004.jpg]
