# Supplementary material for: Novel Alternative Splice Variants of Mouse Cdk5rap2
Source: PLoS One. 2015 Aug 31;10(8):e0136684. doi: 10.1371/journal.pone.0136684 (PMC4556188; doi:10.1371/journal.pone.0136684)
Supplement: S1 Table — (DOCX) [file pone.0136684.s005.docx]

**S1 Table. Primer sequences used for PCR validation of positive ESC clones**

| **PCR** | **Primer Name** | **Primer sequences** | **PCR product size** |
| --- | --- | --- | --- |
| 5' external | Ef | GGCAGTAATAGAAAGTTGCCCTGGAG | 4 kb |
|  | Veclox3 | GTTATCTGCAGGTCGACCTTAAGCT |  |
| loxP | Lf | ctgtctgagttactgacatgtga | 0.22 kb WT  0.32 kb cKO |
|  | Lr | gatgcccctgcgggcaacttcc |  |
| 3' external | Nf | AGGGGCTCGCGCCAGCCGAACTGTT | 4.5 kb |
|  | Er | TAATTGGCCTCAGTGGCCGCTGCAGAATTTGCTCTCAAAAATG |  |
