# Supplementary material for: Novel Alternative Splice Variants of Mouse Cdk5rap2
Source: PLoS One. 2015 Aug 31;10(8):e0136684. doi: 10.1371/journal.pone.0136684 (PMC4556188; doi:10.1371/journal.pone.0136684)
Supplement: S2 Table — (DOCX) [file pone.0136684.s006.docx]

**S2 Table. Neo Southern: Digestions used to validate the 5’ and 3’ insertion**

| **Probe** | **Name** |  | **Genomic DNA digest** | **WT allele (kb)** | **Targeted Allele (kb)** |
| --- | --- | --- | --- | --- | --- |
| Neo | 5’ arm first digest |  | EcoRV | / | 13.4 |
|  | 5’ second digest |  | NheI | / | 9.2 |
|  | 3’ arm first digest |  | Afl II | / | 14.3 |
