# Supplementary material for: Novel Alternative Splice Variants of Mouse Cdk5rap2
Source: PLoS One. 2015 Aug 31;10(8):e0136684. doi: 10.1371/journal.pone.0136684 (PMC4556188; doi:10.1371/journal.pone.0136684)
Supplement: S3 Table — (DOCX) [file pone.0136684.s007.docx]

**S3 Table. External Probe Southern: Digestions used to validate with 5’ and 3’ probes**

| **Probe** | **Name** |  | **Genomic DNA digest** | **WT allele (kb)** | **Targeted Allele (kb)** |
| --- | --- | --- | --- | --- | --- |
| 5' external | first digest |  | Ase I | 8.1 | 10.1 |
|  | second digest |  | Sca I | 7.6 | 8.7 |

Primers for probe synthesis:

5’ probe

CCGCAATTTAATTTCTTAGTATCTG

CCATGCAATCAAACTGTATGAGAGA
