# Supplementary material for: Novel Alternative Splice Variants of Mouse Cdk5rap2
Source: PLoS One. 2015 Aug 31;10(8):e0136684. doi: 10.1371/journal.pone.0136684 (PMC4556188; doi:10.1371/journal.pone.0136684)
Supplement: S4 Table — (DOCX) [file pone.0136684.s008.docx]

**S4 Table. Cdk5rap2 mutant mice used in experiments**

| **Classification** | **Genotypes** | |
| --- | --- | --- |
|  | Cdk5rap2 LoxP | hCMV Cre |
| WT | -/- | - |
|  | -/- | + |
|  | +/- | - |
| het KO | +/- | + |
|  | +/- | - * |
| hom KO | +/+ | + |
|  | +/+ | - * |

*Het KO and hom KO, hCMV Cre^-^ mutant mice (constitutive knockout mice) were generated through breeding Cdk5rap2 LoxP^+/-^ / hCMV Cre^+^ mice among each other, followed by rearing out the hCMV Cre through crossing in hCMV Cre^-^ mice.
