# Supplementary material for: Novel Alternative Splice Variants of Mouse Cdk5rap2
Source: PLoS One. 2015 Aug 31;10(8):e0136684. doi: 10.1371/journal.pone.0136684 (PMC4556188; doi:10.1371/journal.pone.0136684)
Supplement: S5 Table — (DOCX) [file pone.0136684.s009.docx]

**S5 Table. Primer sequences for genotyping**

| **Position** | **Primer** | **Sequence** |
| --- | --- | --- |
| Er | 4659 | CGTAGGAGGCAAAAGCAGGCCG |
| Lf | 4654 | GGTCCCAGCAAGGCCTGTGAGC |
| Lf | 4655 | TGTGGCAGCAGAGGTTGTGTGGC |
| Lf | 4661 | GCAGGGCAGGCGGATGGTAAAT |
| Lr | 4662 | GGGATGAACCCAAAACTTACACAGGA |
| Lxr | 4656 | GCATACATTATACGAAGTTATCTGCA |
| Del1 | Deleter 1 | CGCCATCCACGCTGTTTTGACC |
| Del2 | Deleter 2 | CAGCCCGGACCGACGATGAAG |
