# Supplementary material for: Novel Alternative Splice Variants of Mouse Cdk5rap2
Source: PLoS One. 2015 Aug 31;10(8):e0136684. doi: 10.1371/journal.pone.0136684 (PMC4556188; doi:10.1371/journal.pone.0136684)
Supplement: S6 Table — (DOCX) [file pone.0136684.s010.docx]

**S6 Table. PCR fragments expected size (bp)**

| **Region analyzed** | | **Primers** | **Position** | | **cKO allele (LoxP)** | **KO allele (KO)** | **Wild Type allele (WT)** | |
| --- | --- | --- | --- | --- | --- | --- | --- | --- |
| Presence of the distal LoxP | | 4661 / 4662 | Lf / Lr | | 473 | --- | 379 | |
| Excision of the floxed exon (i.e. knock out) | | 4655 / 4659 | Lf / Er | | 1287 | 504 | 1087 | |
| LoxP specific PCR | | 4654 / 4656 | Lf / Lxr | | 316 | 316 | --- | |
| **Region analyzed** | **Primers** | | | **Position** | | **Cre^+^** | | **Cre^-^** |
| Cre | Deleter 1 / deleter 2 | | | Del1 / Del 2 | | 489 | | --- |
