# Supplementary material for: Novel Alternative Splice Variants of Mouse Cdk5rap2
Source: PLoS One. 2015 Aug 31;10(8):e0136684. doi: 10.1371/journal.pone.0136684 (PMC4556188; doi:10.1371/journal.pone.0136684)
Supplement: S7 Table — (DOCX) [file pone.0136684.s011.docx]

**S7 Table. Primer and probe sequences for qPCR**

| **Primer / Probe** | **Sequence** | **Notes** |
| --- | --- | --- |
| mCdk5rap2-F | TCAGAGGCGTTGGGTGAGA |  |
| mCdk5rap2-R | GGATCAACAAGCCCGTCTTC |  |
| mCdk5rap2 probe | FAM-CAACAGGCCACTCACCTCTCATTCCC-TMR |  |
| Exon3 ins (Cdk5rap2) | CGGAACATGAAGGACTTTGAAA | Binds to exon 3 |
| Exon3 del (Cdk5rap2) | TGGGAAATGGAGCAAATCAC | Binds only, if exon 3 is deleted |
| Cdk5rap2-A | GGAGGCTTTGACGAGCAAC |  |
| Cdk5rap2 probe | FAM-TCAATGTTCGTCTTGTAGATGTGCTCGGT-BBQ |  |
| F-71bp-insert (Cdk5rap2) | GGTTGCACACTTCCTTTGGT |  |
|  |  |  |
| mHprt-F | ATCATTATGCCGAGGATTTGGAA |  |
| mHprt-R | TTGAGCACACAGAGGGCCA |  |
| mHprt probe | FAM-TGGACAGGACTGAAAGACTTGCTCGAGATG-TMR. |  |
