# Supplementary material for: Novel Alternative Splice Variants of Mouse Cdk5rap2
Source: PLoS One. 2015 Aug 31;10(8):e0136684. doi: 10.1371/journal.pone.0136684 (PMC4556188; doi:10.1371/journal.pone.0136684)
Supplement: S8 Table — (DOCX) [file pone.0136684.s012.docx]

**S8 Table. Primer sequences for sequencing of human *CDK5RAP2***

| **Primer** | **Sequence** | **Species** |
| --- | --- | --- |
| F ex2 | CTTCACCCTGTTCTGCCAAG | mouse |
| R ex4 | TTCTCCTTCTTCAGCTCAGTGAT | mouse |
| R ex7 | CCAGAGCCAGAGTCATCTCC | mouse |
| mCdk5rap2 F Ex1 | ACTGCCGGGGAACCAGT | mouse |
| mCdk5rap2 R Ex11 | GGTGGAGTCCGTAGTGACCT | mouse |
| hCDK5RAP2 Ex2 F | GCTGGGTTGGGAAATGGTC | human |
| hCDK5RAP2 Ex5 R | GGAGTTCCCGCTTCAGACTT | human |
