# Supplementary material for: Novel Alternative Splice Variants of Mouse Cdk5rap2
Source: PLoS One. 2015 Aug 31;10(8):e0136684. doi: 10.1371/journal.pone.0136684 (PMC4556188; doi:10.1371/journal.pone.0136684)
Supplement: S9 Table — (DOCX) [file pone.0136684.s013.docx]

**S9 Table. Primer sequences for 5’-RACE**

| **Primer** | **Sequence** | **Localization** |
| --- | --- | --- |
| RACE SP1 Ex14 | GTCTGGTGACTGCTGAGAACAC | Exon 14 |
| RACE SP2 Ex12 | CAACCTATGGTTCTCAGTGCTC | Exon 12 |
| RACE SP3 Ex9 | CTCCTTCTGCCATTCCTCAG | Exon 9 |
